# Low Protein Diets Supplemented With Alpha-Ketoglutarate Enhance the Growth Performance, Immune Response, and Intestinal Health in Common Carp (*Cyprinus carpio*)

**DOI:** 10.3389/fimmu.2022.915657

**Published:** 2022-06-02

**Authors:** Di Wu, Ze Fan, Jinnan Li, Yuanyuan Zhang, Qiyou Xu, Liang Wang, Liansheng Wang

**Affiliations:** ^1^Key Laboratory of Aquatic Animal Diseases and Immune Technology of Heilongjiang Province, Heilongjiang River Fisheries Research Institute, Chinese Academy of Fishery Sciences, Harbin, China; ^2^School of Life Science, Huzhou University, Huzhou, China; ^3^AHP Application Research Institute, Weifang Addeasy Bio-Technology Co., Ltd, Weifang, China

**Keywords:** alpha-ketoglutarate, common carp, growth performance, immune response, intestinal health

## Abstract

To investigate the effects of alpha-ketoglutarate (AKG) supplementation in a low protein (LP) diet on the growth performance, immune response, and intestinal health of common carp (*Cyprinus carpio*), 600 carp were randomly divided into five dietary groups: a normal protein (NP) diet containing 32% crude protein, an LP diet formulated with 28% crude protein, and LP with AKG at 0.4%, 0.8%, and 1.2% (dry matter). After an 8-week trial period, the results demonstrated that an LP diet led to a decrease in performance, immune response, and intestinal barrier function. Compared with the LP group, the final body weight and weight gain rate in the LP+0.4% AKG group were significantly higher, the feed conversion ratio was significantly decreased with the addition of 0.4% and 0.8% AKG. The supplementation with 0.4% and 0.8% AKG markedly increased the activities of T-SOD and GSH-Px, as well as the expression levels of *GPX1a* and *GPX1b* relative to the LP group, whereas the MDA content was significantly decreased in the LP+0.4% AKG group. In addition, the expression levels of tight junctions including *claudin-3*, *claudin-7*, *ZO-1*, and *MLCK* were significantly up-regulated in the LP+0.4% AKG group, and the relative expression levels of the pro-inflammatory factors *IL-1β* and *IL-6α* were significantly lower with the addition of 0.4%, 0.8%, and 1.2% AKG. Moreover, the abundance of *Proteobacteria* in the LP+0.4% AKG group was lower than that in the LP group, and the abundance of *Firmicutes* and *Fusobacteria* was higher at the phylum level. The abundance of *Citrobacter* in the LP+0.4% AKG group was decreased compared to the LP group, while the abundance of *Aeromonas* was increased at the genus level. In short, the effects of AKG on the intestinal health of the common carp were systematically and comprehensively evaluated from the perspectives of intestinal physical barrier, chemical barrier, biological barrier, and immune barrier. We found that an LP diet supplemented with 0.4% AKG was beneficial to the growth performance and intestinal health of common carp.

## 1 Introduction

In recent years, the shortage of protein resources and the rising price are forcing the aquatic industry to confront a huge limitation (1). An effective approach to alleviate the current tension is exploring the feasibility of low protein (LP) feeds and reduce the demand for protein sources (2). However, protein deficiency, characterized by LP intake, weakens the immune system and aggravates inflammatory diseases (3–5). The nutritional therapeutic strategy has been considered to be an effective approach for improving growth and intestinal health (6).

Alpha-ketoglutarate (AKG) is a central molecule in the tricarboxylic acid cycle (Krebs cycle), it can be rapidly converted into glutamate through transamination by glutamate dehydrogenase, and further into glutamine (7–9). AKG has a great effect on promoting the absorption and metabolism of amino acids. The addition of AKG could enhance energy status and muscle mass by activating mechanistic target of rapamycin (mTOR) signaling pathway in piglets (10). Previous studies have also demonstrated that dietary AKG supplementation improved the growth performance and protein deposition of juvenile hybrid sturgeon (*Acipenser schrenckiif* × *A.baeriio*) (11). Additionally, as a precursor of glutamine, AKG supplementation showed beneficial effects on protecting cells from reactive oxygen species (ROS) such as free radicals and peroxides (12). In a model of piglets, dietary AKG supplementation alleviated the decrease of total superoxide dismutase (T-SOD) and glutathione peroxidase (GSH-Px) activities, and the increase of malondialdehyde (MDA) content induced by lipopolysaccharide (13). Similarly, AKG supplementation also increased energy storage and improved hepatic anti-oxidative capacity in ducks (14). However, the effects of dietary AKG on the intestinal immunity and the intestine health of animals, especially in fish, has not been sufficient studied.

The intestine is an important organ for digestion, absorption and growth of fish (15). Intestinal health is closely related to mucosal immunity and structural integrity (16, 17), and the damage to intestinal immune function and structural integrity reduces the growth of fish (18), which is correlated with intestinal physical barrier, chemical barrier, biological barrier, and immune barrier. Therefore, improving the immune function and intestinal barrier of fish is crucial to ensuring their intestinal health and promoting growth. Over the past few years, our laboratory has explored several studies on AKG regulating the intestinal health and immune function of fish, especially in common carp (*Cyprinus carpio*). A previous study has shown that AKG played a critical role in the intestinal immunity, and AKG could significantly increase the thickness of intestinal muscle layer and fold height and significantly enhance the activities of intestinal digestive enzymes (19). In addition, it has been confirmed that AKG alleviated the decline in intestinal function caused by the inflammatory response after *Aeromonas hydrophila* infection (20). However, the mechanism by which AKG regulates the immune function and structural integrity of the intestine health in an LP diet remains unclear in fish.

Based on reported benefits in previous studies, we hypothesized that AKG supplementation has a positive effect on the immune response and intestinal barrier in common carp fed with an LP diet. Our objective was to investigate how combining an LP diet with AKG might influence the growth performance, immune responses, and intestinal health of common carp. The data obtained here will help us further understand the mechanism through which AKG improves performance and immune response in fish, and provide a scientific basis for using AKG in commercial practice.

## 2 Materials And Methods

### 2.1 Animals

Heilongjiang River Fisheries Research Institute of the Chinese Academy of Fishery Sciences provided the common carp. Following the guidelines for animal experiments guidelines for the care and use of laboratory animals of Heilongjiang River Fisheries Research Institute of Chinese Academy of Fishery Sciences, the studies in animals were reviewed and approved by the Committee for the Welfare and Ethics of Laboratory Animals of Heilongjiang River Fisheries Research Institute of Chinese Academy of Fishery Sciences.

### 2.2 Experimental Design and Feeding Trial

Common carp were randomly divided into five groups (1): normal protein (NP) diet with 32% crude protein (2), LP diet with 28% crude protein, and LP diet with (3) 0.4% AKG supplementation (LP+0.4% AKG) (4), 0.8% AKG supplementation (LP+0.8% AKG), and (5) 1.2% AKG supplementation (LP+1.2% AKG). These inclusion levels were selected according to the use of AKG in a previous study (21). The formulation of the diet was based on the nutrient necessities of common carp according to the guidelines of the NRC (2011). **Table 1** shows the ingredients of the diets. Soybean meal, wheat meal, and fish meal were used as dietary protein sources. Soybean oil, soybean lecithin and fish oil were used as dietary lipid sources. The ingredients were ground into fine powder through an 80 mesh sieve. Each diet was individually weighed and blended in a mixer and then homogenized with water and extruded into pellets using a machine pelletizer. The pellets were air-dried in an oven at 65°C for 4 h and stored in airtight containers at -20°C until used for feeding.

**Table 1:** Composition and nutrients content of the experimental diets (g/kg, dry matter).

Items	NP	LP	LP+0.4% AKG	LP+0.8% AKG	LP+1.2% AKG
Soybean meal^a^	480.00	480.00	480.00	480.00	480.00
Wheat middling^a^	300.00	360.00	360.00	360.00	360.00
Fish meal^a^	100.00	40.00	40.00	40.00	40.00
Soybean oil	30.00	30.00	30.00	30.00	30.00
Soybean lecithin	10.00	10.00	10.00	10.00	10.00
Fish oil	10.00	10.00	10.00	10.00	10.00
Sodium hydroxy cellulose	10.00	10.00	10.00	10.00	10.00
Choline chloride	5.00	5.00	5.00	5.00	5.00
Monocalcium phosphate	25.00	25.00	25.00	25.00	25.00
Vitamin premix^b^	3.00	3.00	3.00	3.00	3.00
Trace mineral premix^c^	2.00	2.00	2.00	2.00	2.00
Cellulose	17.30	12.80	8.80	4.80	0.80
AKG^d^	0.00	0.00	4.00	8.00	12.00
Lysine	3.00	5.60	5.60	5.60	5.60
Methionine	2.20	2.90	2.90	2.90	2.90
Threonine	2.50	3.70	3.70	3.70	3.70
Proximate composition (g/kg dry matter)					
Crude protein^e^	321.42	287.72	285.26	288.39	283.65
Crude lipid^e^	62.89	54.13	53.19	56.96	56.12

a Soybean meal, obtained from Hefeng feedstuffs Co., Ltd, crude protein 440.00 g/kg, crude lipid 15.00 g/kg. Wheat middling, obtained from Jinlongyu Grain, Oil and Food Co. Ltd, crude protein 130.00 g/kg, crude lipid 12.00 g/kg. Fish meal, obtained from Hefeng feedstuffs Co., Ltd, crude protein 600.00 g/kg, crude lipid 48.50 g/kg.

b The vitamin premix provided the following per kg of the diet: VA 8,000 IU, VC 500 mg, VD_3_ 3,000 IU, VE 60 mg, VK_3_ 5 mg, VB_2_ 30 mg, VB_6_ 15 mg, VB_12_ 0.5 mg, choline chloride 5,000 mg, nicotinic acid 175 mg, D-biotin 2.5 mg, inositol 1,000 mg, folic acid 5 mg, pantothenic acid 50 mg.

c The mineral premix provided the following per kg of the diet: Zn 25 mg, Cu 3 mg, Fe 25 mg, Mn 15 mg, I 0.6 mg, Co 0.1 mg, Se 0.4 mg.

d AKG (alpha-ketoglutarate, Sigma-Aldrich) with a purity of 98%.

e Crude protein and crude lipid were measurement value.

Prior to the experiment, the carp were fed with a basal diet for 2 weeks for acclimation. Carp (initial body weight = 5.00 ± 0.74 g) were randomly selected and distributed into tanks (0.7 m × 0.7 m × 0.8 m) after 24 h of starvation with 30 fish per tank and 4 tanks per group. Water qualities were measured throughout the experimental period and maintained at water temperature of 23-25°C, pH 6.8-7.3, dissolved oxygen more than 6 mg/L and photoperiod 12D:12L. The carp were fed 5 g/100 g body weight of the allocated diet and feeding rates were adjusted every two weeks after weighing. Fish were fed to apparent satiation three times daily at 08:00 and 13:00 and 17:00 for 8 weeks, and one-third of the water was renewed per day.

### 2.3 Sampling Procedure

At the end of the experiment, all fish from each tank were harvested, counted and weighed (g). Then, 9 fish from each tank were anesthetized using buffered 2-Phenoxyethanol (30 mg/L with purity ≥99%, Code No: 77699, Sigma-Aldrich, Shanghai, China). Afterward, the intestinal tissue and luminal digesta were collected from each group (3 fish per tank in quadruplicate). The intestines of 3 fish per tank (a total of 12 fish per group) were dissected, weighed, and homogenized in PBS buffer and centrifuged at 4500 g at 4°C for 20 min. The supernatant was collected and stored at -20°C for further assays of biochemical analysis and antioxidant status. The intestines of 3 fish per tank were collected in cryogenic vials and stored at -80°C for quantitative real-time PCR (qRT-PCR) analysis. The rest intestines of 3 fish per tank were placed in a centrifuge tube with fixative fluid and stored at 4°C for transmission electron microscopy (TEM) characterization. The luminal digesta was collected after 7 h of feeding in cryogenic vials and stored at -80°C for sequencing analysis.

### 2.4 Growth Performance

The parameters of growth performance including weight gain rate (WGR), feed conversion ratio (FCR), and specific growth rate (SGR) were calculated as follows:


$$\text{Weight gain rate }\left(\text{WGR, }\%\right)=\frac{W2-W1}{W1}\times 100$$

$$\text{Specific growth rate }\left(\text{SGR, }\%/\text{day}\right)=\frac{\text{Ln }W2-\text{Ln }W1}{T}\times 100$$

$$\text{Feed conversion ration }\left(\text{FCR}\right)=\frac{\text{feed intake}\;\left(\text{g}\right)}{W2-W1}$$

where *W1* and *W2* are the initial and final weights, respectively, and *T* is the number of days in the feeding period.

### 2.5 Biochemical Analysis

The lipase (LPS, Cat. No. A054-1-1), amylase (AMS, Cat. No. C016-1-1), and trypsin (Try, Cat. No. A080-2-2) contents in tissue homogenate were measured using commercially available kits (Nanjing Jiancheng Bioengineering Institute, Nanjing, China) according to the manufacturer’s protocol. Each assay was performed in triplicate.

### 2.6 Antioxidant Status

Catalase (CAT, Cat. No. A007-1-1), superoxide dismutase (SOD, Cat. No. A001-3-1), glutathione peroxidase (GSH-Px, Cat. No. A005-1-2), and malondialdehyde (MDA, Cat. No. A003-1-2) activities in tissue homogenate were measured according to commercial kits (Nanjing Jiancheng Bioengineering Institute) following the protocols provided by the supplier.

### 2.7 qRT-PCR Analysis

Procedures of total RNA isolation, reverse transcription, and qRT-PCR were similar to our previous study (20). Briefly, total RNA was isolated using a TRIzol method (Code No: 9109, TaKaRa, Dalian, China) according to the manufacturer’s instructions. RNA purity was determined by measuring the OD ratio at 260/280 nm using a NanoDrop 2000 (Thermo Scientific, Waltham, USA). Total RNA samples with a high RNA ratio (A260/A280 = 1.8-2.0) were used for cDNA synthesis as calculated (1 ng/μL) for each reaction. For the PCR amplification, 2 μL of cDNA, 2 μL of primers, and SYBR Green Master Mix (Code No: RR047A, TaKaRa, Dalian, China) in a total volume of 20 μL were used following the manufacturer’s protocol. cDNAs were subjected to a thermal cycling condition in ABI 7500 real-time PCR machine (ABI, Applied Biosystems, USA) as follows: initial denaturation at 95°C for 30 s, then 40 cycles of 95°C for 5 s and 62°C for 34 s. At the end of the last cycle, 95°C for 15 s, 60°C for 1 min, 95°C for 30 s, and 60°C for 15 s. A list of primers used in the present study is provided in **Table 2**. The cycle at which the sample fluorescence faced a predetermined threshold (Ct) considerably beyond the background was used for determining the quantity of each sample. Samples were tested in duplicate and the mean was used for further analysis. The housekeeping gene (β-actin) was applied for normalizing all the data of the sample and control groups according to the preliminary experiment (data not shown). The critical threshold (Ct) quantities of target genes were normalized using the 2^-ΔΔCt^ method as described by Livak and Schmittgen (22).

**Table 2:** Real-time PCR primer sequences.

Target gene	Primer sequence (5’→3’)	Length	Accession number
TLR4^a^	F: TGTCGCTTTGAGTTTGAAT	19	NW_017540541.1
	R: TCCAGAATGATGATGATGATC	21	
MyD88^b^	F: AAGAGGATGGTGGTAGTCA	19	LN590716.1
	R: GAGTGCGAACTTGGTCTG	18	
NF-κB^c^	F: TATTCAGTGCGTGAAGAAG	19	LN590678.1
	R: TATTAAAGGGGTTGTTCTGT	20	
TNF-α^d^	F: AAGTCTCAGAACAATCAGGAA	21	AJ311800
	R: TGCCTTGGAAGTGACATT	18	
IL-1β^e^	F: AACTTCACACTTGAGGAT	18	KC008576
	R: GACAGAACAATAACAACAAC	20	
IL-6^f^	F: GACCAGCAGGTACGTCTCAACAC	23	LN590906.1
	R: TCCTTCATACGCCGTCATGTTCAC	24	
IL-8^g^	F: AAACTGAGAGTCGACGCATTG	21	EU011243.1
	R: TTTTCAATGACCTTCTTAACCCAG	24	
IL-10^h^	F: GCCAGCATAAAGAACTCG	18	JX524550.1
	R: CCAAATACTGCTCGATGT	18	
TGF-β2^i^	F: GGGACATCATCGCCATCT	18	U66874.1
	R: TGACATTCTCGGCAGGGT	18	
claudin-1	F: GACAACATCRTSACVGCHCAG	21	LN598389.1
	R: CMYTYCCRAACTCATACCT	19	
claudin-3	F: GCACCAACTGTATCGAGGATG	21	LN590711.1
	R: GGTTGTAGAAGTCCCGAATGG	21	
claudin-7	F: CTTCTATAACCCCTTCACACCAG	23	LN591006.1
	R: ACATGCCTCCACCCATTATG	20	
claudin-11	F: TCGGAAGTGAACCAGAAAGC	20	LN590700.1
	R: GAAGCCAAAGGACATCAAGC	20	
occludin	F: ATCGGTTCAGTACAATCAGG	20	LN590695.1
	R: GACAATGAAGCCCATAACAA	20	
ZO-1^j^	F: GCCTGCCTACACTCAACCACAAC	23	LN590708.1
	R: CTGCTTCGGCTGGAGGAGGAG	21	
MLCK^k^	F: CGATGGTGGCAGTGCTGTGAC	21	LN590717.1
	R: GACTCTTGGCTCGGTTCGCTAAC	23	
β-actin	F: GATCGGCAATGAGCGTTTCC	20	M24113.1
	R: ACGGTGTTGGCATACAGGTC	20	

a TLR4, toll-like receptor 4.

b MyD88, myeloid differentiation factor 88.

c NF-κB, nuclear factor kappa-B.

d TNF-α, tumor necrosis factor-α.

e IL-1β, interleukin-1β.

f IL-6, interleukin-6.

g IL-8, interleukin-8.

h IL-10, interleukin-10.

i TGF-β2, transforming growth factor-β2.

j ZO-1, zonula occludens-1.

k MLCK, myosin light chain kinases.

### 2.8 TEM Characterization

Intestinal samples were placed in a 2.5% glutaraldehyde solution (Code No: 1.04239, Sigma-Aldrich, Shanghai, China) overnight to avoid light. Subsequently, the samples were continuously treated with a graded ethanol series (50%, 70%, 90%, and 100%) for 8 min, 100% ethanol for 10 min, a mixture (v/v = 1:1) of 100% ethanol and acetone (Code No: 179124, Sigma-Aldrich, Shanghai, China) for 15 min, and absolute acetone for 15 min. The samples were then immersed in a 1:1 mixture of absolute acetone and epoxy resin (Code No: 45345, Sigma-Aldrich, Shanghai, China) for 30 min and pure epoxy resin overnight. Then, the samples were sectioned with an ultramicrotome, stained with uranyl acetate and lead citrate, and observed using a Hitachi H-7650 transmission electron microscope (Hitachi, Toyko, Japan).

### 2.9 Analyses of Intestinal Microbiota

#### 2.9.1 DNA Extraction, PCR Amplification, and Illumina MiSeq Sequencing

Microbial genomic DNA was extracted from intestinal contents by using an OMEGA D4015 Stool DNA Kit (Omega, USA) following the manufacturer’s instruction. The quantity and purity of the extracted genomic DNA were estimated by NanoDropND-2000 spectrophotometer (Thermo Fisher Scientific, Waltham, USA) and agarose gel electrophoresis, respectively. Specific primers with a barcode were synthesized according to the specified sequencing region. TransGen AP221-02: TransStart FastPFU DNA Polymerase (ABI Geneamp ^®^ 9700) was used for PCR with three replicas for each sample. PCR products from the same sample were mixed and detected by 2% agarose gel electrophoresis and recovered by gel-cutting using the AxyPrepared DNA Gel Recovery Kit (Cat. No. AP-GX-250, Axygen, Shanghai, China) and eluted by Tris-HCl (Cat. No. 20490, Thermo Fisher Scientific, Shanghai, China) and 2% agarose electrophoresis. The PCR product was quantified using the Quantifluor™-ST Blue Fluorescence Quantitative System (Promega Inc., Beijing, China) and then mixed in proportion to the required sequencing volume for each sample. All purified amplicons were sequenced using high-throughput sequencing on the Illumina Miseq sequencing platform at the laboratories of Shanghai Majorbio Bio-pharm Technology Co. Ltd., (Shanghai, China). Paired-end (PE) reads obtained by Miseq sequencing were first spliced according to overlap relationship, and the sequence quality was controlled and filtered simultaneously. Operational taxonomic unit (OTU) cluster and species taxonomy analyses were performed after the samples were distinguished.

#### 2.9.2 Sequence Data Processing and Bioinformatics Analysis

Raw sequences of all tested samples were processed using Uparse (version 7.1). All sequence reads were trimmed and assigned to each sample based on their barcodes. Sequences with high quality (length > 300 bp, without the ambiguous base “N”, an average base quality score > 30) were used for further analysis. Assignment of OTUs were completed at 97% identity. A Venn analysis was performed in R software to identify unique and common OTUs among carp in the NP, LP and LP+0.4% AKG diet groups. The alpha-diversity metrics of intestinal microbiota, including Ace index, Chao index, Good’s coverage, Shannon index, Simpson index, and Sobs were calculated using Mothur 1.30.2. For the beta-diversity metrics, the principal coordinate analysis (PCoA) was performed using OTUs for each sample through the QIIME pipeline. Nonmetric multidimensional scaling (NMDS) and analysis of molecular variance (AMOVA) were performed to evaluate the overall differences in bacterial community structure among groups based on the Bray-Curtis distance. All-against-all strategies were used to compare significant differences among groups.

### 2.10 Statistical Analysis

The data obtained was expressed as mean ± standard error of the mean (SEM). Results were analyzed statistically by one-way ANOVA followed by Tukey’s honestly significant difference test as a *post hoc* test. All parameters were analyzed by SPSS software Version 22.0 (SPSS Inc., Chicago, IL, USA). *P* values less than 0.05 were considered statistically significant. Data visualization was performed by using the GraphPad Prim 5.0 (GraphPad Inc., La Jolla, CA, USA).

## 3 Results

### 3.1 Effects of LP Diet Supplemented with AKG on the Growth Performance of Common Carp

**Figure 1:**
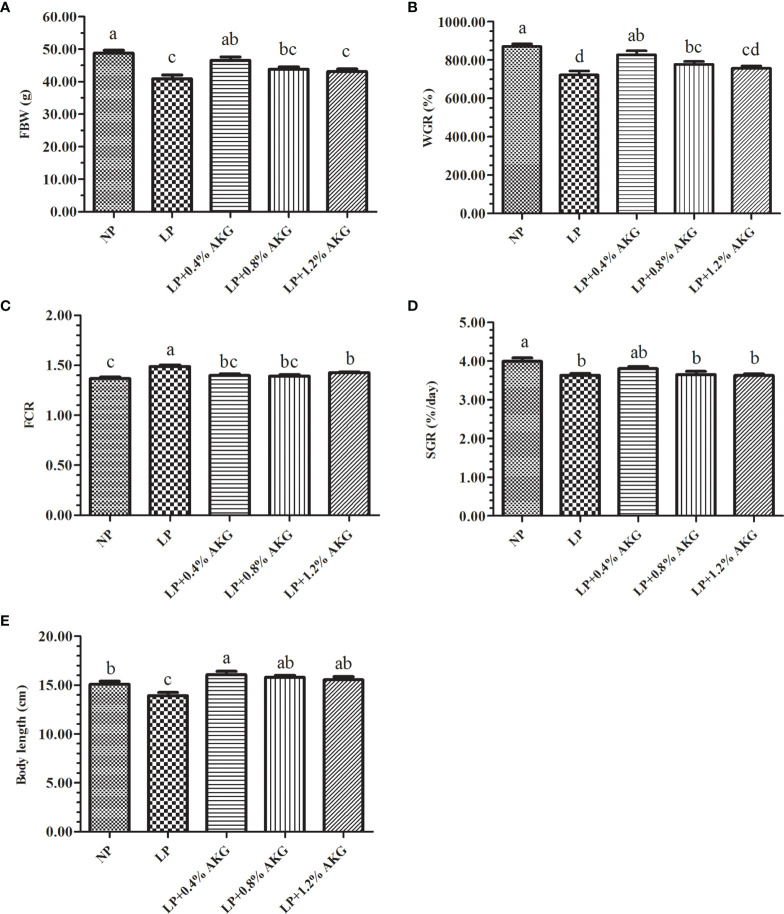
Growth performance of common carp (*Cyprinus carpio*) fed with diets containing normal protein (NP), low protein (LP), and different concentrations of alpha-ketoglutarate (AKG) after 8 weeks. **(A)** FBW, final body weight (g); **(B)** WGR, weight gain rate (%); **(C)** FCR, feed conversion ratio; **(D)** SGR, specific growth rate (%/day); **(E)** Body length (cm). The initial body weight of common carp was 5.00 ± 0.74 g. Values are expressed as the mean ± SEM (n = 4). Values marked with different letters indicate significant differences (*P* < 0.05) between groups.

The effects of LP diets supplemented with AKG on the growth performance are shown in **Figure 1**. The results showed that the FBW, WGR, SGR, and body length in the LP group were significantly lower than those in the NP group (*P* < 0.05), and the FCR was significantly increased (*P* < 0.05). Compared with the LP group, the FBW and WGR in the LP+0.4%AKG group were significantly increased (*P* < 0.05), and the FCR was significantly decreased (*P* < 0.05). There were no significant differences in FBW, WGR, SGR and FCR between the NP and LP+0.4%AKG groups (*P* > 0.05). Meanwhile, the LP+0.8% AKG group significantly increased the WGR and decreased the FCR compared with the LP group (*P* < 0.05), whereas the FBW, WGR, and SGR in the LP+0.8% AKG and LP+1.2% AKG groups were significantly lower than those in the NP group (*P* < 0.05). Fish fed on diets in the LP with 0.4%, 0.8%, and 1.2% AKG groups showed a significant increase in body length compared with the LP group (*P* < 0.05).

### 3.2 Effects of LP Diet Supplemented With AKG on Immune Digestive Enzymes in the Intestinal Chemical Barrier of Common Carp

As demonstrated in **Table 3**, the LPS contents in the NP and LP+0.4% AKG groups were significantly higher than that in the LP, LP+0.8% AKG and LP+1.2% AKG groups (*P* < 0.05). The highest AMS content was observed in the LP group, with significant differences compared with the NP, LP+0.4%AKG, LP+0.8% AKG, and LP+1.2% AKG groups (*P* < 0.05). There was no significant difference in the Try contents among all groups (*P* > 0.05).

**Table 3:** Digestive enzymes s of common carp (*Cyprinus carpio*) fed with diets containing normal protein (NP), low protein (LP), and different concentrations of alpha-ketoglutarate (AKG) after 8 weeks.

	NP	LP	LP+0.4% AKG	LP+0.8% AKG	LP+1.2% AKG
Try^a^ (U/gprot)	26.01 ± 8.27	20.31 ± 6.64	30.80 ± 9.98	28.87 ± 6.29	23.25 ± 2.48
LPS^b^ (U/gprot)	14.67 ± 1.32^a^	8.60 ± 1.60^b^	13.32 ± 2.58^a^	8.87 ± 1.95^b^	6.22 ± 1.62^b^
AMS^c^ (U/dL)	3699.10 ± 220.47^b^	4100.30 ± 189.03^a^	3549.31 ± 343.98^b^	3610.83 ± 335.68^b^	3574.73 ± 146.59^b^

Values are expressed as the means ± SEM (n = 4), and different superscript letters in the same line are significantly different (P < 0.05).

a Try, trypsin.

b LPS, lipase.

c AMS, amylase.

### 3.3 Effects of LP Diet Supplemented With AKG on Antioxidant Capacity in the Intestine of Common Carp

**Figure 2:**
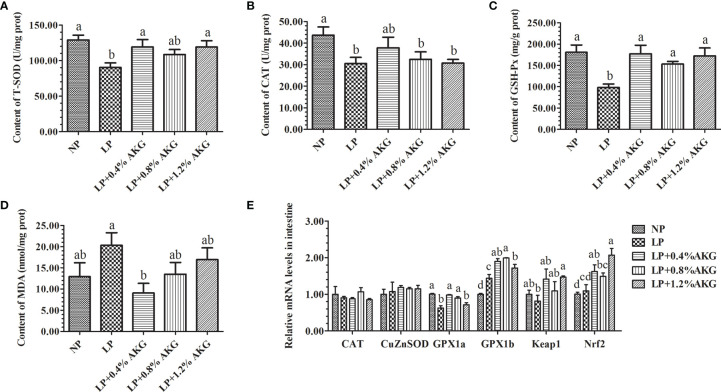
Antioxidant capacity in the intestine of common carp (*Cyprinus carpio*) fed with diets containing normal protein (NP), low protein (LP), and different concentrations of alpha-ketoglutarate (AKG) after 8 weeks. **(A)** T-SOD, total superoxide dismutase (U/mg·prot); **(B)** CAT, catalase (U/mg·prot); **(C)** GSH-Px, glutathione peroxidase (mg/g·prot); **(D)** MDA, malondialdehyde (mmol/mg·prot); **(E)** Relative expressions of antioxidant-related genes *via* Nrf2/Keap1 signaling pathway. Antioxidant-related genes includes catalase (CAT), copper, zinc superoxide dismutase (CuZnSOD), glutathione peroxidase 1a (GPx1a), glutathione peroxidase 1b (GPx1b), Kelch-like-ECH-associated protein 1 (Keap1) and NF-E2-related factor 2 (Nrf2). Values are expressed as the mean ± SEM (n = 4). Values marked with different letters indicate significant differences (*P* < 0.05) between groups.

Results of the antioxidant capacity of common carp are presented in **Figures 2A** **–D**. Antioxidant capacity represented by T-SOD, GSH-Px, and MDA levels indicated that fish fed on LP with 0.4%, 0.8%, and 1.2% AKG did not show any significant variations relative to the NP group (*P* > 0.05). Compared with the NP group, T-SOD, and GSH-Px values were significantly decreased in the LP group (*P* < 0.05). T-SOD values in the LP with 0.4% and 1.2% AKG groups were increased significantly relative to the LP group (*P* < 0.05), and fish fed on diets in the LP with 0.4%, 0.8%, and 1.2% AKG groups showed a significant increase in GSH-Px compared with the LP group (*P* < 0.05). Additionally, CAT values did not show a significant difference among all five groups (*P* > 0.05). There was a significant decrease in the MDA values of the LP+0.4% AKG group relative to the LP group (*P* < 0.05).

The expression levels of antioxidant-related genes (*CAT*, *CuZnSOD*, *GPX1a*, *GPX1b*, *Keap1*, and *Nrf2*) in the intestine are presented in **Figure 2E**. No significant (*P* > 0.05) differences were observed in the gene expression of *CAT* and *CuZnSOD* among the NP, LP, and LP with 0.4%, 0.8%, and 1.2% AKG groups. There were no significant changes in the expression of the *GPX1a* gene (*P* > 0.05) among the NP, LP+0.4% AKG, and LP+0.8% AKG groups, whereas a significantly lower level of expression was observed in the LP followed by the LP +1.2% AKG group (*P* < 0.05). Moreover, *GPX1b* gene expression was higher in the LP group (*P* < 0.05). With increasing dietary AKG levels, the expression of the *Keap1* and *Nrf2* genes was first down-regulated, then up-regulated, peaking in the group fed with 1.2% AKG. Additionally, the gene expression of *Keap1* was significantly (*P* < 0.05) up-regulated compared with the LP group, and *Nrf2* gene expression in the LP+1.2% AKG group showed a significant (*P* < 0.05) up-regulated in relation to the NP, LP, and LP+0.8% AKG groups.

### 3.4 Effects of LP Diet Supplemented With AKG on the NF-κB Signaling Pathway in the Intestinal Immunological Barrier of Common Carp

**Figure 3:**
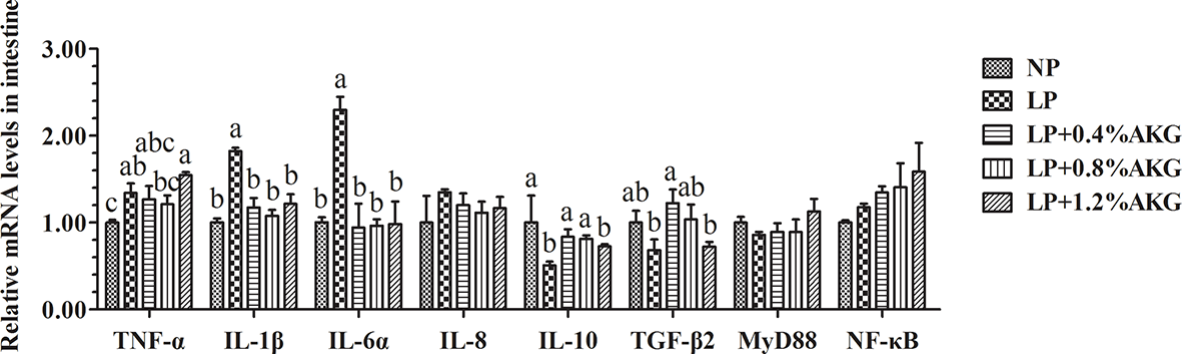
Relative expressions of inflammatory-related genes *via* NF-κB signaling pathway in the intestinal immunological barrier of common carp (*Cyprinus carpio*) fed with diets containing normal protein (NP), low protein (LP), and different concentrations of alpha-ketoglutarate (AKG) after 8 weeks. Inflammatory-related genes includes tumor necrosis factor-α (TNF-α), interleukin-1β (IL-1β), interleukin-6α (IL-6α), interleukin-8 (IL-8), interleukin-10 (IL-10), transforming growth factor-β2 (TGF-β2), myeloid differentiation factor 88 (MyD88) and nuclear factor kappa-B (NF-κB). Values are expressed as the mean ± SEM (n = 4). Values marked with different letters indicate significant differences (*P* < 0.05) between groups.

As shown in **Figure 3**, *TNF-α* gene expression did not show significant changes among the NP, LP+0.4% AKG, and LP+0.8% AKG groups (*P* > 0.05), whereas a significantly higher expression level was observed in the LP+1.2% AKG followed by the LP group (*P* < 0.05). Naturally, *IL-1β* and *IL-6α* gene expression had a significantly higher expression in the LP group (*P* < 0.05). The expression of the *IL-1β* and *IL-6α* genes in the LP with 0.4%, 0.8%, and 1.2% AKG groups were significantly lower than those in the LP group (*P* < 0.05). No significant changes were observed in the expression of *IL-1β* and *IL-6α* genes among the NP, and LP with 0.4%, 0.8%, and 1.2% AKG groups (*P* > 0.05). Additionally, *IL-10* and *TGF-β2* gene expression did not show significant changes among the NP, LP+0.4% AKG, and LP+0.8% AKG groups (*P* > 0.05), whereas its lowest expression was observed in the LP followed by the LP+1.2% AKG group (*P* < 0.05). Results additionally demonstrated that no significant differences were observed in the gene expression of *IL-8*, *MyD88*, and *NF-κB* among all groups (*P* > 0.05).

### 3.5 Effects of LP Diet Supplemented With AKG on Tight Junctions in the Intestinal Physical Barrier of Common Carp

**Figure 4:**
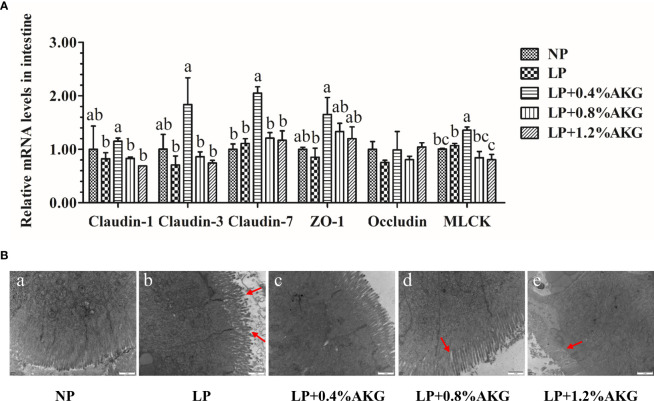
Tight junctions in the intestine of common carp (*Cyprinus carpio*) fed with diets containing normal protein (NP), low protein (LP), and different concentrations of alpha-ketoglutarate (AKG) after 8 weeks. **(A)** Relative expressions of tight junction-related genes *via* MLCK signaling pathway. Tight junction-related genes includes claudin-1, claudin-3, claudin-7, zonula occludens-1 (ZO-1), occludin and myosin light chain kinases (MLCK). Values are expressed as the mean ± SEM (n = 4). Values marked with different letters indicate significant differences (*P* < 0.05) between groups. **(B)** TEM micrographs. Scale bar of TEM is 1 μm.

All data resulting from the gene expression of tight junctions are presented in **Figure 4A**. A general increase in the expression of tight junction genes, as indicated by *claudin-1*, *claudin-3*, *claudin-7*, *ZO-1*, and *MLCK*, was found in response to 0.4% AKG supplementation. The expression levels of *claudin-1*, *claudin-3*, and *MLCK* in the LP+0.4% AKG group were significantly (*P* < 0.05) up-regulated compared with the LP, LP+0.8% AKG, and LP+1.2% AKG groups, whereas no significant differences were shown between the NP and LP+0.4% AKG groups (*P* > 0.05). In regards to *claudin-7*, AKG supplemented at 0.4% allowed the highest value of expression levels compared to other groups including NP, LP, LP+0.8% AKG, and LP+1.2% AKG (*P* < 0.05). The gene expression level of *ZO-1* in the LP+0.4% AKG group was significantly up-regulated compared to the LP group (*P* < 0.05) although it was not significantly up-regulated compared to the NP group (*P* > 0.05). No significant differences were observed in the gene expression of *occludin* among all the groups (*P* > 0.05).

The more intuitive results observed by TEM are shown in **Figure 4B**. The NP group had intestines with normal morphology with intestinal villi closely arranged, whereas we observed damage to the intestines, such as villous atrophy and shedding, in the LP groups. The LP group had the most severe intestinal villi damage. Interestingly, the addition of 0.8% or 1.2% AKG to the LP group partly improved the histological morphology of the intestine of common carp. However, the most obvious improvement in intestinal villi and compactness was observed in the LP+0.4% AKG group.

### 3.6 Effects of LP Diet Supplemented With AKG in the Intestinal Microbiological Barrier of Common Carp

**Figure 5:**
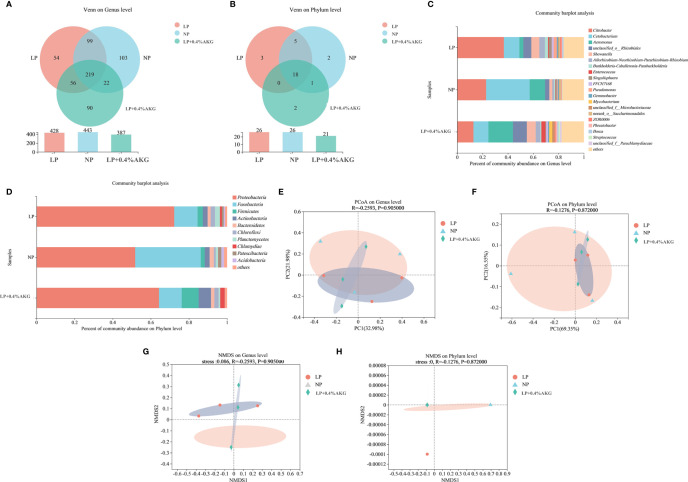
Differences between gut microbiota communities of common carp (*Cyprinus carpio*) fed with diets containing normal protein (NP), low protein (LP), and 0.4% concentrations of alpha-ketoglutarate (AKG) after 8 weeks. **(A)** Venn diagram on Genus level; **(B)** Venn diagram on Phylum level; **(C)** Relative abundance of gut microbes on Genus level; The ordinate is the sample name, the abscissa is the proportion of species in the sample, the columns with different colors represent different species, and the length of the columns represents the proportion of species. **(D)** Relative abundance of gut microbes on Phylum level. **(E)** Principal coordinate analysis (PCoA) plot based on unweighted UniFrac distance (PCoA1: 32.98% and PCoA2: 21.98% of the explained variance) on Genus level; **(F)** PCoA plot based on unweighted UniFrac distance (PCoA1: 69.35% and PCoA2: 16.55% of the explained variance) on Phylum level; **(G)** Nonmetric multidimensional scaling (NMDS) analysis of bacterial community composition in carp of treatments on Genus level; each symbol represents a sample (circles: LP, triangles: NP, diamond: LP+0.4%AKG). The ellipse was drawn around the position of each group of samples. **(H)** NMDS analysis of bacterial community composition in carp of treatments on Phylum level.

A total of 502116 clean reads were determined from all experimental groups. Alpha diversity analysis showed that no significant change (*P* > 0:05) was found in the Ace, Chao, Coverage, Shannon, Simpson and Sobs indexes (**Table 4**). As shown in **Figure 5A**, 219 OTUs were shared in each group of samples at the genus level, and the number of unique OTUs in the NP, LP, and LP+0.04% AKG groups was 103, 54, and 90, respectively. At the phylum level, 18 OTUs were shared in each group of samples. The number of unique OTUs in the NP, LP, and LP+0.04% AKG groups was 2, 3, and 2, respectively (**Figure 5B**).

**Table 4:** The alpha-diversity indexes of the bacterial community of common carp (*Cyprinus carpio*) fed with diets containing normal protein (NP), low protein (LP), and 0.4% concentrations of alpha-ketoglutarate (AKG) after 8 weeks.

	Ace	Chao	Coverage	Shannon	Simpson	Sobs
NP	274.93 ± 138.80	251.33 ± 155.60	1.00 ± 0.00	2.12 ± 1.62	0.36 ± 0.30	212.67 ± 181.68
LP	283.28 ± 56.65	287.89 ± 44.20	1.00 ± 0.00	2.49 ± 1.24	0.28 ± 0.30	252.00 ± 65.48
LP+0.4%AKG	263.35 ± 64.90	253.52 ± 68.09	1.00 ± 0.00	2.65 ± 0.55	0.16 ± 0.12	212.67 ± 80.31

Three intestine from each of the three tanks were tested. Values are expressed as the means ± SEM (n = 3), and different superscript letters in the same row are significantly different (P < 0.05).

As can be seen from **Figure 5C**, the abundance of *Citrobacter* and in the LP group was higher than that in the NP group, and the abundance of *Cetobacterium* and *Aeromonas* in the LP group was lower than that in the NP group. Compared with the LP group, the abundance of *Citrobacter, Cetobacterium*, and *Pseudomonas* was decreased in LP+0.4%AKG group, whereas the abundance of *Aeromonas* was increased. Supplementation with 0.4% AKG reduced the abundance of *Citrobacter* and *Pseudomonas* in the LP diet. *Pseudomona*s belonging to *Proteobacteria* showed the same trend as the decrease in the abundance of *Proteobacteria*. As shown in **Figure 5D**, the abundance of *Proteobacteria*, *Chloroflexi*, and *Planctomycetes* in the LP group was higher than that in the NP group, and the abundance of *Fusobacteria* and *Bacteroidetes* in the LP group was lower than that in the NP group. The abundance of *Proteobacteria* in the LP+0.4% AKG group was lower than that in the LP group, and the abundance of *Firmicutes* and *Actinobacteria* in the LP+0.4% AKG group was higher than that in the LP group. As shown in **Figure 5E**, there was some difference in clustering degree between LP+0.4%AKG group and LP group, although this difference did not appear at the phylum level (**Figure 5F**). As can be seen from **Figures, 5G, H**, there was a significant separation difference in the composition of intestinal flora at the genus and phylum levels between the LP group and the NP group, and the addition of 0.4% AKG reduced the difference.

## 4 Discussion

### 4.1 Growth Performance

The purpose of this study was to supplement an LP diet (4% lower than NP) with AKG while applying current production practices to improve growth performance and intestinal immune barrier function including digestive enzymes, antioxidant capacity, inflammation-related gene expression, tight junction proteins and microbial communities of the common carp. In general, the growth of fish is closely related to the deposition of protein. Previous studies have demonstrated that the growth performance such as average daily gain and feed efficiency were significantly improved by supplementing AKG in piglets (23). In this study, compared with the results from the LP group, we noted that dietary supplementation with AKG tended to improve FBW, WGR, and FCR. Our data also demonstrated that the addition of 0.4% AKG to the LP group improved the growth performance of common carp significantly, which is consistent with the findings in piglets (24). It is possible that dietary AKG supplementation promotes intestinal development and increases energy-digestibility (25), while reducing populations of harmful bacteria and modifying their metabolites within the gastrointestinal tract, which was confirmed in the later results. Studies have shown that this response may partly be associated with an increase in amino acid and energy digestibilities (26). In addition, AKG is an intermediate of the citric acid cycle and a ubiquitous amine collector in tissues, and AKG has been reported to stimulate AMPK phosphorylation and the oxidation of energy substrates in the intestinal mucosa, thereby inactivating one of its downstream target enzymes, acetyl-CoA carboxylase (ACC) (27). This inactivation converts acetyl-CoA to malonate Acyl-CoA, an inhibitor of fatty acid oxidation and a substrate for fatty acid synthesis that enhances ATP supply, thereby increasing body fat storage and weight gain. When AKG supplementation reached a certain amount, increasing the level did not further improve the growth performance of the carp, the reason for this needs further study.

### 4.2 Chemical Barrier

Intestinal digestive enzyme activities are closely related to the growth and development of fish, especially carp, and the absorption of nutrients (28). Trypsin and lipase activities reflect the secretion capacity of intestinal digestive enzymes. Preliminary results have shown that the improvement of the intestinal absorption function of fish can be achieved by adding an appropriate amount of AKG to the diet (29). AKG can generate glutamine by reacting AKG with the glutamine (Gln) metabolic axis. Studies have confirmed that Gln can increase the functional surface area of the intestinal mucosa and promote the absorption of nutrients in the intestine (24). Therefore, intestinal digestive enzyme activity was positively correlated with its growth performance. The results of this study showed that different levels of AKG had a positive effect on the digestive enzyme activities of common carp, which was consistent with the above conclusion. The addition of 0.4% AKG in this study significantly increased the activities of intestinal trypsin and lipase, indicating that AKG could promote intestinal material transport, and improve intestinal digestion and absorption capacity, which provided a guarantee for the improvement of feed protein and lipid digestibility in common carp.

### 4.3 Antioxidant Responses

A large number of free reactive oxygen species (ROS) are released after the oxidase system is stimulated externally. Excessive free radicals cause protein structure damage, destroy cell structure and function, and damage the intestinal mucosal barrier (30). Studies have shown that the LP levels led to a significant increase in the level of oxidative stress in humans (31), rats (32) and piglets (33). In the present study, compared with the control, T-SOD, CAT, and GSH-Px levels were significantly attenuated in an LP diet, while 0.4% and 0.8% AKG supplementation elevated T-SOD and GSH-Px levels. These findings indicated that AKG reduced the lipid peroxidation level of common carp in the LP diet and provided the carp with a stronger ability to resist oxygen free radicals, positively impacting the growth performance and health of the fish, which might be attributed to the conversion from AKG to glutamine and finally to GSH (34). MDA is the end product of lipid peroxidation, which indicates an imbalance of the antioxidant system in fish (35). In this study, MDA content in an LP diet was significantly reduced with 0.4% AKG supplementation. This indicated that AKG had a certain antioxidant effect on the intestine of carp, which was beneficial in the protection of the intestinal mucosal barrier.

The Nrf2 signaling pathway is a key pathway of cellular anti-oxidative stress (36). Normally, Nrf2 binds rapidly to the cytoplasmic inhibitor protein Keap1 through the ubiquitin-proteasome pathway to initiate the expression of a variety of downstream protective genes, including antioxidant protease genes (37). Studies have shown that Nrf2 regulated the expression of antioxidant enzyme genes such as *SOD1* and *GSH-Px* in the European eel (*Anguilla anguilla*) (38). In this study, the LP diets supplemented with different levels of AKG, especially at the 0.4% levels, increased the expression levels of *Nrf2*, *Keap1* and antioxidant enzyme genes to varying degrees, and the change in the trend of their corresponding antioxidant enzyme activities was consistent. Therefore, supplementation with an appropriate amount of AKG could enhance the antioxidant stress ability of carp. This was consistent with previous studies that dietary AKG has a positive effect on neutrophil oxidative radical production (39). The up-regulated expression of antioxidant enzymes may be related to the activation of the Nrf2/Keap1 signaling pathway by AKG to promote up-regulated *Nrf2* gene expression in the intestine of carp. On the whole, the above results confirmed that dietary supplementation with 0.4% AKG contributed to enhancement of antioxidant defense system enzymes activities in common carp. Supplementation with 0.4% AKG may play beneficial role in maintaining ROS equilibrium with free radical scavenging. It could be speculated that AKG can exerted an antioxidative defense through the Nrf2/Keap1 signaling pathway, while improving the activities of SOD and CAT, and nonenzymatic GSH levels, thereby reducing the levels of MDA.

### 4.4 Immune Barrier

Numerous studies have shown that dietary protein levels are closely related to immune function. Cytokines are important signal molecules for regulating immune cells, and their expression can represent the immune response status. *TNF-α*, *IL1-β*, and *IL6-α* are the major inflammatory cytokines produced and released by immune cells, which induce synergistic effects of other inflammatory mediators (40). In this study, it was found that an LP diet significantly increased the expression of intestinal inflammatory factors, suggesting that the common carp had an increased risk of intestinal inflammation. Studies have confirmed that the expression level of *TNF-α* was increased after an LP diet in piglets (41), which is consistent with our results. Correspondingly, the expression levels of anti-inflammatory cytokines *IL-1*0 and *TGF-β2* were decreased in the LP group compared with the NP group, indicating that an LP diet did indeed trigger an inflammatory response. AKG has been briefly mentioned in several studies as a key nutrient factor in improving the intestinal immune system (42). In piglets, AKG alleviated the inflammatory response of piglets caused by an LP diet (43). Our previous study also showed that dietary AKG decreased the mRNA expression levels of pro-inflammatory cytokines including *TNF-α*, *IL-1β* and *IL-8* (20). Similar to the above results, this study found that the expression levels of *IL-1β* and *IL6-α* were significantly decreased with dietary AKG supplementation. At the same time, the expression levels of *IL-10* and *TGF-β2* were up-regulated in the LP group supplemented with 0.4% AKG. These observations could be partly attributed to AKG serving as a precursor of glutamine and glutamate in tissues with a positive effect that improves immunity during an inflammatory response (44), which protected the intestinal mucosal structure and barrier function by restricting the production of cytokines and inflammatory responses.

### 4.5 Physical Barrier

As the most important form of cell-to-cell connection, tight junctions have the mechanical function of holding epithelial cells together, while strengthening cell-to-cell connections and making cells less vulnerable to damage (45). The expression level and distribution structure of claudins directly affect the structure and function of tightly connected membrane proteins (46). Relative to the LP group, AKG increased the expression levels of intestinal *claudin-1*, *claudin-3*, *claudin-7* and *ZO-1*, suggesting that AKG supplementation in an LP diet had a positive protective effect on intestinal tight junction proteins in common carp. Our previous studies showed that the mRNA expression levels of tight junctions including *claudin-1*, *claudin-3*, *claudin-7*, and *claudin-11* were up-regulated with AKG supplementation after infection with *A. hydrophila* (20). These observations were partly attributed to AKG supplementation improving glutamine synthesis activity and concentrations of Gln and glutamic acid in the intestines (29). Gln has been shown to promote protein synthesis and intestinal development in fish (47). Dietary Gln supplementation showed significantly higher expression levels of *claudin 3*, *claudin 4*, *occludin*, and *ZO-1* in the intestines of turbot (48). It has been shown that the MLCK signaling pathway was one of the important pathways regulating intestinal epithelial tight junctions, and the activation and nuclear transfer of NF-κB was an important mechanism of MLCK transcription and synthesis. Continuous activation of NF-κB induces MLCK transcription, which in turn activates downstream signaling pathways, leading to the enhancement of tight junctions (49). In our study, compared with the LP group, the expression level of MLCK was significantly up-regulated after 0.4% AKG supplementation, suggesting that the MLCK signaling pathway was activated. Studies have found that MLCK regulated transmembrane proteins and associated proteins through non-kinase activity to activate the tight junction barrier, which controls intestinal permeability (50). Based on these results, AKG may improve the intestinal function and health of common carp by regulating the MLCK signaling pathway and maintaining tight junction s, including claudins and ZO-1 proteins through indirect immune-mediation and re-distribution.

To observe the effect of AKG on the intestinal tight junction protein more intuitively, we performed an intestinal projection by using TEM. In the images of intestinal morphology, damaged intestinal tight junction and loose and incomplete villi occurred in the LP group. AKG supplementation alleviated the damage caused by the LP diet. This result showed that AKG improved intestinal immunity and enhanced the digestion and absorption capacity of the intestine, which had a positive impact on the growth performance of carp. In brief, the LP diet increased the permeability of the intestinal epithelium and made the intestinal cells vulnerable to damage. AKG alleviated this process, thereby improving intestinal barrier function.

### 4.6 Biological Barrier

Microbial community composition is a key determinant of host health in the context of metabolism and inflammation. Alpha diversity results showed that dietary LP altered intestinal microflora diversity in carp, which was consistent with the results in pigs (51), grass carp (*Ctenopharyngodon idellus*) (52), and tilapia (*Oreochromis niloticus*) (53). Studies have shown that there was a competitive relationship between microliters in the digestive tract, and increased microbial competition led to the reduction of species and quantity (54). In human studies, it was found that the decrease of intestinal microbial diversity was related to intestinal diseases (55), indicating that the LP diet may result in carp that are in a substandard state of health and more vulnerable to intestinal diseases. In addition, Liu et al. (56) believed that the instability of fish intestinal flora diversity was related to metabolism, indicating that an LP diet may also lead to metabolic disorder in carp.

*Proteobacteria* are microbial markers of gut dysbiosis. Dysbiosis during metabolic disorders is often accompanied by an increase in *Proteobacteria*. Recent studies have shown that metabolic disturbances in gut microbial communities can be characterized by *Proteobacteria* abundance. The increase in the number of *Proteobacteria* in the carp gut caused by feeding an LP diet marked an imbalance in the carp gut microbial community. The study found that short-term unstable intestinal flora, especially a community dominated by *Proteobacteria*, caused increased susceptibility to chronic colitis in rats (57). This may also be the reason why the expression levels of inflammatory factors in the carp intestinal tract were up-regulated after feeding an LP diet. Studies have shown that the intestinal flora of healthy fish secrete digestive enzymes such as protease and lipase, which played a key role in promoting the digestion and absorption of nutrients in fish (58). This was reflected by the rise in Try, LPS and AMS in our study when AKG diets were fed over the entire experimental period. In addition, our study is the first to take an *in-vivo* approach to examine the impact that AKG has on gut composition in common carp. At the phylum level, our bacteriological results indicated that the dietary supplementation of AKG increased the populations of intestinal *Firmicutes*, *Fusobacteria*, and *Bacteroidetes* but decreased the proportion of *Proteobacteria*. Comprising the largest proportion of both mouse and human gut microbiomes, *Firmicutes* are also involved in energy-recycling (59). *Firmicutes* could effectively ingest the heat in the organic matter from dietary and endogenous origins and promote the storage of fat in the host body (60). AKG may provide an energy source for intestinal microbiota that helps improve the growth of *Firmicutes* populations by maintaining optimum gastrointestinal functions. At the genus level, our study also demonstrated that AKG supplementation decreased *Citrobacter* levels compared to in the LP group. In mice, the pathogen *Citrobacter* disrupts intestinal mucosa and causes inflammation and diarrhea (61). On the contrary, the content of *Aeromonas* increased, suggesting that AKG could improve the community structure in the intestinal tract of carp, which was also verified by NMDS analysis. This suggested that the changes in gut microbiome structure were due to its adaptation to the intestinal environment created by adding AKG to the diet, and AKG could promote intestinal health in carp.

In conclusion, this study systematically evaluated the effects of different concentrations of AKG on the growth performance, immune response, and intestinal health of common carp under LP conditions. The effects of AKG on chemical, physical, biological, and immune barriers and antioxidant responses were evaluated in common carp fed an LP diet. Our research data showed that supplementing LP feed with 0.4% AKG effectively alleviated the intestinal damage caused by LP, and improved the growth performance, antioxidant capacity, and intestinal health of common carp. In general, our conclusions provided an advanced theoretical basis for the further application of LP feeds. Simultaneously, it also provides a new strategy for nutritional intervention strategy of supplementing AKG in an LP diet to alleviate intestinal inflammation of common carp.

## Data Availability Statement

The datasets presented in this study can be found in online repositories. The names of the repository/repositories and accession number(s) can be found below: https://www.ncbi.nlm.nih.gov/, PRJNA839892.

## Ethics Statement

The animal study was reviewed and approved by the Committee for the Welfare and Ethics of Laboratory Animals of Heilongjiang River Fisheries Research Institute of Chinese Academy of Fishery Sciences.

## Author Contributions

DW carried out the whole process of the experiments, analysed experimental data, and wrote the manuscript. LSW and QX designed the study. ZF, JL and YZ reviewed the manuscript. LW provided technical assistance. All authors contributed to the article and approved the submitted version.

## Funding

This work was supported by the Central Public-interest Scientific Institution Basal Research Fund, CAFS (2022XT0402), the Central Public-interest Scientific Institution Basal Research Fund, HRFRI (HSY202206Q, HSY202002M), the National Natural Science Foundation of China (31972800, 31802305), the China Agriculture Research System of MOF and MARA.

## Conflict of Interest

Author LW is employed by Weifang Addeasy Bio-Technology Co., Ltd.

The remaining authors declare that the research was conducted in the absence of any commercial or financial relationships that could be construed as a potential conflict of interest.

## Publisher’s Note

All claims expressed in this article are solely those of the authors and do not necessarily represent those of their affiliated organizations, or those of the publisher, the editors and the reviewers. Any product that may be evaluated in this article, or claim that may be made by its manufacturer, is not guaranteed or endorsed by the publisher.
